# Pulmonary hypertension in connective tissue diseases, new evidence and challenges

**DOI:** 10.1111/eci.13453

**Published:** 2020-12-05

**Authors:** Madelon C. Vonk, Els Vandecasteele, Arie P. van Dijk

**Affiliations:** ^1^ Department of the Rheumatic diseases Radboud University Nijmegen Medical Centre Nijmegen the Netherlands; ^2^ Department of Cardiology Gent University Gent Belgium; ^3^ Department of Cardiology Radboud University Nijmegen Medical Centre Nijmegen the Netherlands

**Keywords:** connective tissue disease, pulmonary hypertension, systemic lupus erythematosus, systemic sclerosis

## Abstract

Pulmonary arterial hypertension is a lethal complication of different connective tissue diseases such as systemic sclerosis, mixed connective tissue disease and systemic lupus erythematosus. Although the treatment possibilities for patients with pulmonary arterial hypertension have increased in the last two decades and survival of patients with idiopathic pulmonary arterial hypertension has improved, the latter is not the case for patients with pulmonary arterial hypertension associated with connective tissue disease. In this narrative review, we review recent literature and describe the improvement of early diagnostic possibilities, screening modalities and treatment options. We also point out the pitfalls in diagnosis in this patient category and describe the unmet needs and what the focus of future research should be.

## 1 INTRODUCTION

Pulmonary hypertension (PH) is a progressive disease characterized by an elevated pulmonary arterial pressure and pulmonary vascular resistance. As a consequence of the elevated pulmonary arterial pressure, patients are at risk of right heart failure and death. Patients with connective tissue diseases (CTD) such as systemic sclerosis (SSc), mixed connective tissue disease (MCTD) and systemic lupus erythematosus (SLE) may develop PH as a complication of their autoimmune disease.^1^ Also in patients suffering from CTDs, the occurrence of PH has a major impact on quality of life and prognosis. In the last years, our knowledge on risk factors, screenings modalities and treatment options has increased and prognosis has improved. In this narrative review, PH in CTD is discussed, with the focus on pulmonary arterial hypertension (PAH), taken up‐to‐date knowledge on screening, early diagnosis, treatment options and prognosis into account.

## 2 CLINICAL PRESENTATION AND DIAGNOSIS

Pulmonary hypertension is a severe clinical condition in which loss and obstructive remodelling of the pulmonary vascular bed is responsible for the rise of pulmonary arterial pressure (PAP) and pulmonary vascular resistance (PVR), resulting in progressive right heart failure and functional decline.^2^ Patients with CTD such as SSc, MCTD and SLE are at risk of PH; with PAH being the most progressive form of PH, but also other forms of PH occur and CTD patients often suffer from overlap of different forms of PH.^1^ The first signs and symptoms of PAH are generally vague and nonspecific. Patients experience fatigue, dyspnoea on exertion, weakness and light headedness. Those symptoms are often ascribed to the CTD itself or to having a low physical fitness. More severe symptoms such as (near) syncope, angina and/or oedema only occur after extensive pulmonary vasculopathy has developed. The diagnostic approach in patients with a clinical suspicion for PH should include physical examination, laboratory work‐up, pulmonary function testing including forced vital capacity (FVC) and diffusion capacity for carbon monoxide (DLCO), echocardiography, high‐resolution chest computed tomography scan and ventilation/perfusion lung scanning as the differential diagnosis of PAH should be taken into account.^3^ Blood tests are not useful for the diagnosis of PH but may help distinguish forms of PH and prognosis, such as an elevated N‐terminal pro‐BNP (NT‐pro‐BNP) level.^3^ Pulmonary function tests including total lung capacity, FVC and DLCO reveal a mild restrictive component with a marked reduction of DLCO in PAH.^3^ Echocardiography shows a dilated and hypertrophic right ventricle with signs of pressure overload such as systolic septal flattening. Using the regurgitant velocity of tricuspid regurgitation and pulmonary regurgitation and the shape of the velocity curve of the right ventricular outflow tract velocity, systolic, diastolic and mean pulmonary arterial pressures can be estimated. Together with the echocardiographic estimation of right atrial pressure, cardiac index and left atrial pressures, a fairly accurate impression of pulmonary haemodynamics can be obtained.^4^ Ventilation/perfusion lung scanning is the preferred diagnostic tool for chronic embolic disease.^3^ The high‐resolution chest computed tomography may show parenchymal lung disease.^3^ The gold standard to diagnose all forms of PH is a right heart catheterization performed as described by the European college of cardiologist guidelines.^1^

## 3 CLASSIFICATION

Pulmonary hypertension is diagnosed by right heart catheterization (RHC), revealing a mean pulmonary arterial pressure (PAP) ≥25 mm Hg.^1^, ^5^ With right heart catheterization, PH can be divided into three groups: pre‐capillary PH, isolated post‐capillary PH (IpcPH) and combined pre‐ and post‐capillary PH (CpcPH) by estimation of the pulmonary arterial wedge pressure (PAWP) and pulmonary vascular resistance (PVR). The characteristics are summarized in Table 1.

**Table 1:** Hemodynamic definitions of pulmonary hypertension (PH)^5^

Definition	Characteristics	Clinical groups^a^
Pre‐capillary PH	mPAP ≥ 25 mm Hg PAWP ≤ 15 mm Hg PVR ≥ 3WU	1,3,4 and 5
Isolated post‐capillary PH (IpcPH)	mPAP ≥ 25 mm Hg PAWP > 15 mm Hg PVR < 3 WU	2 and 5
Combined pre‐ and post‐capillary PH (CpcPH)	mPAP ≥ 25 mm Hg PAWP > 15 mm Hg PVR ≥ 3 WU	2 and 5

Abbreviations: mPAP, mean pulmonary arterial pressure; PAWP, pulmonary arterial wedge pressure; PVR, pulmonary vascular resistance; WU, Wood units.

^a^ Group 1: PAH; group 2: PH due to left heart disease; group 3: PH due to lung diseases and/or hypoxia; group 4: due to pulmonary artery obstructions; group 5: PH with unclear and/or multifactorial mechanisms.

Besides this hemodynamic classification, a clinical classification is used. The purpose of clinical classification of PH is to categorize clinical conditions associated with PH based on similar pathophysiological mechanisms, clinical presentation, hemodynamic characteristics and therapeutic management.^5^ The 2018 updated clinical classification of pulmonary hypertension is summarized in Table 2.

**Table 2:** Updated clinical classification of pulmonary hypertension (PH)^5^

1 PAH
1.1 Idiopathic PAH
1.2 Heritable PAH
1.3 Drug and toxin‐induced PAH
1.4 PAH associated with:
1.4.1 Connective tissue disease
1.4.2 HIV infection
1.4.3 Portal hypertension
1.4.4 Congenital heart disease
1.4.5 Schistosomiasis
1.5 PAH with long‐term responders to calcium channel blockers
1.6 PAH with overt features of venous/capillaries (PVOD/PCH) involvement
1.7 Persistent PH of the newborn syndrome
2 PH due to left heart disease
2.1 PH due to heart failure with preserved LVEF
2.2 PH due to heart failure with reduced LVEF
2.3 Vascular heart disease
2.4 Congenital/acquired cardiovascular conditions leading to post‐capillary PH
3 PH due to lung diseases and/or hypoxia
3.1 Obstructive lung disease
3.2 Restrictive lung disease
3.3 Other lung disease with mixed restrictive/obstructive pattern
3.4 Hypoxia without lung disease
3.5 Developmental lung disorders
4 PH due to pulmonary artery obstructions
4.1 Chronic thromboembolic PH
4.2 Other pulmonary artery obstructions
5 PH with unclear and/or multifactorial mechanism
5.1 Haematological disorders
5.2 Systemic and metabolic disorders
5.3 Others
5.4 Complex congenital heart disease

Abbreviations: LVEF, left ventricular ejection fraction; PAH, pulmonary arterial hypertension; PCH, pulmonary capillary haemangiomatosis; PVOD, pulmonary veno‐occlusive disease.

**Figure 1:**
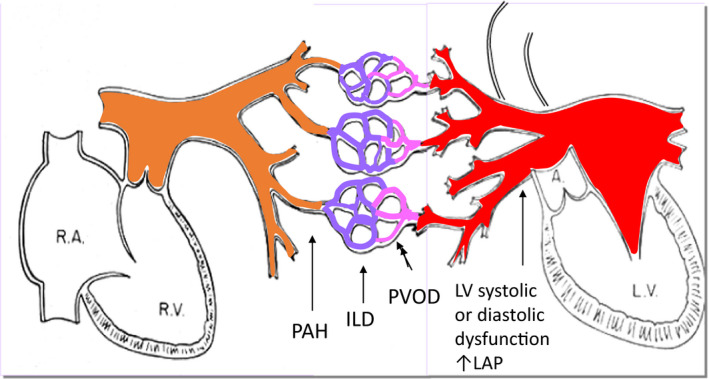
A schematic view of the pulmonary vasculature. A, aorta; ILD, interstitial lung disease; LAP, left artrial pressure; LV, left ventricle; PAH, pulmonary arterial hypertension; PVOD, pulmonary veno‐occlusive disease; RA, right atrium; RV, right ventricle

Different forms of pulmonary hypertension may occur in patients with connective tissue diseases. The most progressive form of PH, group 1 PAH, occurs as a consequence of remodelling and constriction of the pulmonary arteries and arterioles, leading to a progressive increase of the pulmonary vascular resistance and right heart failure.^6^ It is currently accepted that inflammatory mechanisms contribute to PAH genesis and progression, especially in patients with CTD.^7^, ^8^ Inflammatory cell infiltrates have been detected in plexiform lesions from patients with CTD‐associated PAH.^9^ In fact, CTDs are the major cause of PAH, occupying around one‐fourth of the total PAH population.^10^ Group 2 PH, which is PH associated with impaired left heart function, may occur as a consequence of CTD as well. As interstitial lung disease is another common complication of CTDs, especially of SSc and MCTD, group 3 PH may also be contributed to CTD. Group 4 PH, due to chronic embolic disease, may be associated with CTD as well. Of note is pulmonary veno‐occlusive disease which shares the same hemodynamic profile and clinical presentation as other forms of PAH. However, it is associated with a more pronounced venous/capillary involvement and is associated with a poor prognosis, a limited response to PAH therapy and a risk of pulmonary oedema with these treatments.^5^, ^11^ This form of PAH occurs especially in SSc. The localization of the different forms of PH is shown in Figure 1.

## 4 EPIDEMIOLOGY OF PAH IN CONNECTIVE TISSUE DISEASES

Pulmonary arterial hypertension may occur as a complication of various CTDs, such as SSc, MCTD, SLE, primary Sjogren syndrome and (dermato‐) myositis. Among the CTDs, SSc has the highest known PAH prevalence. PAH affects approximately 8%‐12% of patients with SSc and has an incidence of 0.61 cases per 100 patient years.^12^, ^13^ The prevalence of PAH in non‐SSc CTDs remains to be determined due to the lack of large, RHC based cohort studies but is estimated to be lower than in SSc patients.^14^ Based on a nationwide cohort study in Taiwan and a systematic review of the literature, the prevalence of PAH in SLE is <4%.^15^, ^16^ For the other CTDs, the prevalence is estimated to be <1%.^1^

## 5 PROGNOSIS

The prognosis of patients with certain forms of PH has increased with the availability of several vasoactive drugs, better supportive care, earlier diagnosis and earlier treatment. A number of studies and registries have shown a less favourable outcome of patients with PAH associated with SSc compared to those with idiopathic PAH or non‐SSc CTD‐associated PAH.^17^, ^18^ The 3‐year survival rate of PAH associated with SSc is estimated to be only 52%, despite the available targeted treatments.^19^ The survival of SLE‐PAH, studied in a nationwide cohort study in France, is better, with 3‐ and 5‐year overall survival rates of 89.4% and 83.9%, respectively.^20^ In this study, risk factors of death were found to be renal involvement and elevated PVR. In 2 other cohort studies, the presence of anti‐U1‐RNP antibodies was found to be protective factors for survival.^21^, ^22^

## 6 EARLY DIAGNOSIS OF PULMONARY ARTERIAL HYPERTENSION

The symptoms of PAH are nonspecific, especially in CTD patients. Breathlessness, fatigue, weakness, angina and syncope are seen initially during exertion and in advanced cases at rest. Abdominal distension and ankle oedema are signs of progressing right ventricular failure.^1^

**Figure 2:**
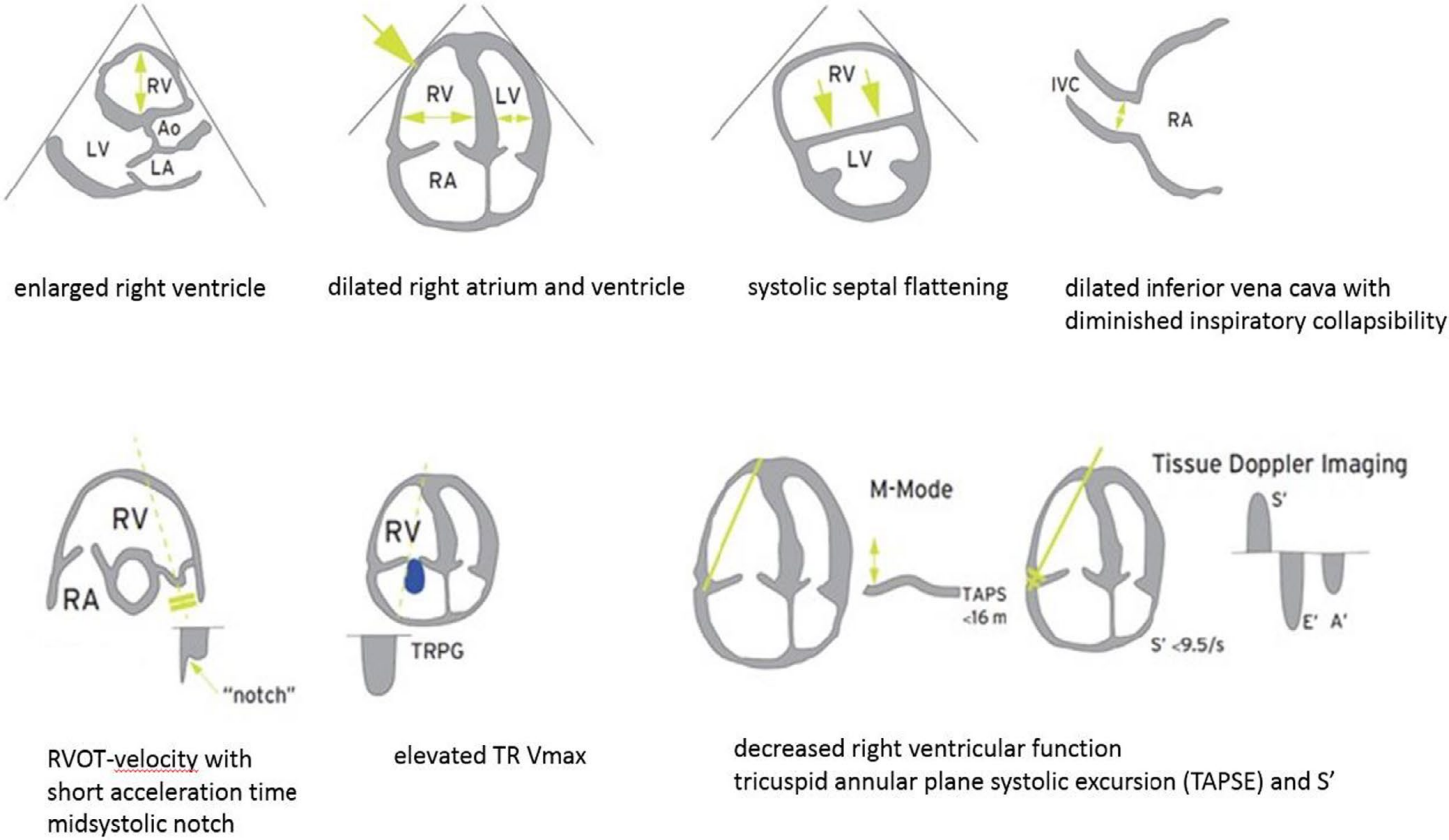
Echocardiography and pulmonary hypertension. A’, active diastolic velocity of the tricuspid annulus; Ao, aorta; E’, early diastolic velocity of the tricuspid annulus; IVC, inferior vena cava; LA, left atrium; LV, left ventricle; RA; right atrium; RV, right ventricle; RVOT, right ventricular outflow tract; S’, systolic velocity of tricuspid annulus; TRPG, tricuspid regurgitation pressure gradient. Adapted from 51

Pulmonary arterial hypertension may occur in both limited cutaneous and diffuse cutaneous SSc, and in patients with a short disease duration and a long disease duration.^23^ In SSc patients without skin involvement yet (‘early’ SSc), only limited date are available in literature.^24^ In a Belgian cohort, none of the 84 included SSc patients without skin involvement were diagnosed with PAH.^25^ Mortality in SSc‐PAH is high, and mortality rates are the highest in the SSc patients who are most severely ill (defined by the New York Heart Association functional class [NYHA FC]) at the moment of PAH diagnosis. In a French registry, the 3‐year survival rate was 30%‐72% in the SSc‐PAH patients diagnosed in NYHA FC III/IV whereas the 3‐year survival rate was up to 80% when diagnosed in NYHA FC II. However, most of the patients were diagnosed in NYHA FC III/IV and only a minority in NYHA FC II at diagnosis.^26^ Systematic screening of SSc patients leads to earlier diagnosis of PAH. Data from a French and an Australian screening programme showed that the SSc patients diagnosed with PAH by screening had lower NYHA FC (≥50% of the included SSc‐PAH patients were in NYHA FC I/II at diagnosis, in both cohorts) and lower pulmonary vascular resistance on right heart catheterization as compared to those who were diagnosed by symptoms.^27^, ^28^ Therefore, early diagnosis is key to improve survival in PAH patients. Screening results in earlier diagnosis and thus earlier treatment. International guidelines recommend screening for PAH in SSc patients from 2009 onwards. Evidence has been accumulating that screening results in improved survival in SSc patients.^1^, ^3^, ^27^, ^29^, ^30^ Different screening methods are used and recommended, based on clinical evaluation, pulmonary function testing, echocardiography, biomarkers or a combination of parameters.^1^, ^3^, ^27^, ^29^, ^30^ For patients with SSc and a disease duration of >3 years and a DLCO a% the DETECT algorithm is used. This DETECT algorithm consists of a 2‐step diagnostic algorithm with clinical, laboratory, lung functional and electrocardiographic parameters in step 1 and echocardiography in step 2, resulting in advice to perform a RHC or not.^31^ This algorithm has proved to reduce the number of missed diagnoses of PAH in SSc compared to using only echocardiography and symptoms.^31^ For all SSc patients, the appropriate screening tools include the 2015 European Society of Cardiology (ESC)/European Respiratory Society (ERS) echocardiographic recommendations (Figure 2), and a combination of a FVC/DLCO > 1.6 and a N‐terminal pro‐BNP > 2 times upper limit of normal. For high‐risk patients (disease duration > 3 years and DLCO < 60% of predicted), the DETECT algorithm can be used as indicator for referral for RHC.^1^, ^3^, ^30^ Current guidelines and recommendations also recognize patients along the SSc spectrum such as MCTD, who have other CTDs but with features of SSc, as needing to be screened similar to patients with SSc.^1^, ^3^, ^30^, ^32^ In other CTDs, systematic screening for PAH is not recommended given its low prevalence. However, for SLE‐associated PAH, several risk factors for PAH have been identified, consisting of longer disease duration of SLE, the presence of interstitial lung disease, the absence of skin rash, the presence of pericardial effusion, the presence of anti‐RNP or anti‐SSA antibodies, low disease activity, low ESR and elevated uric acid.^21^ Careful monitoring of those patients is recommended.

## 7 TREATMENT OF PAH

The treatment of PAH in patients with CTDs is a complex strategy based on initial evaluation of severity and prognostic risk, as well as subsequent response to treatment.^33^, ^34^ Patients with PH are to be referred to expert centres and treated by a multidisciplinary team consisting of rheumatologists, cardiologists and chest physicians as well as specialized nurses. As PAH has a severe impact on daily living and is life‐threatening, psychological, social and emotional support is advocated which is often provided for by the specialized nurses.^35^ General measures for PAH associated with CTD consist of encouragement of physical activity within limits, vaccination against influenza and pneumococcal pneumonia, iron substitution if indicated, the use of diuretics in patients with signs of fluid retention and oxygen when applicable.^1^, ^34^

The use of high‐dose calcium channel blockers (CCB) in CTD‐PAH is not recommended. However, the majority of the SSc patients already take CCB for Raynaud's phenomenon.^36^ In contrast to patients suffering from idiopathic PAH, oral anticoagulant therapy is not recommended.^1^

According to the ESC/ERS guidelines, in patients with CTD‐PAH, the same treatment algorithm as for patients with idiopathic PAH is recommended. Currently approved treatment strategies in PAH target three main pathways: the prostacyclin pathway, the endothelin pathway and the nitric oxide (NO) pathway.^1^, ^34^

### 7.1 Prostacyclin pathway

Prostacyclin analogues bind to the prostacyclin receptor leading to an increase of cyclic adenosine monophosphate (cAMP), resulting in vasodilation, antiproliferative and antithrombotic effects.^1^ Four agents have been licensed by the Food and Drug Administration (FDA) and European Medicines Agency (EMA) for treatment of PAH: epoprostenol (iv), treprostenil (iv, sc, inhaled), iloprost (inhaled) and selexipag, an orally active, selective prostacyclin receptor agonist.

### 7.2 Endothelin pathway

Endothelin‐1 (ET‐1) acts through two receptor subtypes namely ET receptor type A and B. ET‐1 causes vasoconstriction and proliferation of the smooth muscle cells by binding to ET type A and B receptor and increases the NO and prostacyclin production by binding to ET type B receptor.^1^ Bosentan and macitentan are nonselective ET‐1 receptor antagonists (ERA), while ambrisentan is a selective type A ET‐1 receptor antagonist.^1^

### 7.3 Nitric oxide pathway

Reduced nitric oxide (NO) availability is associated with PAH. NO induces vasodilation and inhibits vascular proliferation by increasing production of cyclic guanosine monophosphate (cGMP). Phosphodiesterases (PDEs) are enzymes that inactivate cGMP. Phosphodiesterase five inhibitors (PDE5‐I) (sildenafil and tadalafil) slow the breakdown of cGMP and the soluble guanylate cyclase agonist (riociguat) stimulates cGMP production.^1^

In most of the randomized controlled trials (RCT) on which approval of specific PAH treatment is based, both idiopathic PAH and CTD‐PAH patients are included (up to 30% CTD‐PAH patients). Focusing on CTD‐PAH and SSc‐PAH, there are some general remarks and limitations. RCTs on treatment of PAH in SSc and CTD are scarce and in the RCTs including both idiopathic PAH and CTD‐PAH, subgroup analysis of the CTD‐PAH or SSc‐PAH subgroup are not always performed or the subgroups were not powered to detect statistical significance. An important evolution in the PAH RCT design is the shifting of the primary endpoint from a short‐term correlates such as the six minutes walking test (6MWT) to a long‐term true clinical efficacy measure such as time to clinical worsening or a combined mortality/morbidity endpoint.^37^, ^38^, ^39^ It is known that CTD patients also have many comorbidities such as myositis, arthritis, digital ulcers at the toes and fatigue interfering with the utility of the 6MWT.^34^

Table 3 focuses on the clinical trials or subgroup analyses of clinical trials on treatment of CTD‐PAH and SSc‐PAH. For the first RCTs and subgroup analyses with 6MWT as primary endpoint, lower response rates are seen in CTD‐PAH as compared to idiopathic PAH. However, from 2013 onwards, the SERAPHIN, the GRIPHON and the AMBITION trial used a combined mortality/morbidity primary endpoint, consisting of time from the initiation of treatment to the first event (death from any cause, worsening of PAH and need for additional treatment for PAH, lung transplantation or atrial septostomy).^37^, ^38^, ^39^ In the SERAPHIN and GRIPHON trial, the majority of the patients was already treated with PAH‐specific therapy and in the AMBITION trial initial combination therapy was compared to initiation with monotherapy. In the subgroup analyses of CTD‐PAH patients in all of these three trials, there was a reduction of combined primary endpoint (Table 3).^37^, ^38^, ^39^

**Table 3:** Clinical trials or subgroup analysis of clinical trials on CTD‐PAH and SSc‐PAH

Drug	Reference	Type of study Inclusion criteria	Treatment	Comp	N PAH	N IPAH	N CTD (N SSc‐PAH)	primary endpoint	Effect in CTD‐PAH or SSc‐PAH
Prostacyclins									
Epoprostenol	Badesch 2000^52^	RCT, SSc‐PAH^a^ 6MWT > 50 m NYHA FC II‐IV	Epoprostenol continuous IV	Placebo	111	0	111 (111)	placebo‐corrected Δmedian 6 MWT at week 12	SSc 108 m (95%CI: 55.2 to 180 m)
	Badesch 2009^53^	Open‐label extension of RCT, SSc‐PAH^a^ NYHA FC II‐IV	Epoprostenol continuous IV		97	0	97 (97)	1‐, 2‐, 3‐year survival	SSc 71%, 52% and 48%
Treprostenil	Oudiz 2004^54^	Subgroup analysis of RCT, CTD‐PAH^b^ 6MWT 50‐450 m NYHA FC II‐IV	Treprostenil continuous SC	Placebo	90	0	90 (45)	placebo‐corrected Δ6MWT at week 12	CTD 25 m (*P* = .055)
Selexipag	Gaine 2017 GRIPHON ^39^	Subgroup analysis of RCT, CTD‐PAH^b^ 6MWT 50‐450 m	Selexipag po Titration 200‐1600 µg 2x/d	Placebo	334	0	334 (170)	Combined mortality/morbidity endpoint (median duration of treatment 67 wk)	HR (95%CI) CTD: 0.59 (0.41‐0.85) SSc: 0.56 (0.34‐0.91)
ERA									
Bosentan	Rubin 2002 BREATHE‐1^55^	RCT, IPAH/CTD‐PAH^b^ 6MWT 150‐450 m WHO FC III/IV	Bosentan po 125‐250 mg 2x/d	Placebo	213	150	63(47)	∆6MWT at week 16	SSc 43 m (95%CI: NA, *P* = NS)
	Denton 2006^56^	Subgroup analysis of an open‐label extension of 2 RCTs CTD‐PAH^b^, 6MWT 150‐500 m, WHO FC III/IV	Bosentan po 125‐250 mg 2x/d	Placebo	66	0	66 (52)	placebo‐corrected ∆6MWT at week 12‐16	CTD 22.1 m (95%CI: −32 to 76 m) Survival: 85.9% after 1 y and 73.4% after 2 y of bosentan treatment
Macitentan	Pulido 2013 SERAPHIN ^37^	RCT, IPAH, CTD‐PAH, others^b^, 6MWD > 50 m WHO FC I‐IV	Macitentan po 10 mg/d	Placebo	742	404	224	combined mortality/morbidity primary endpoint (median duration of treatment: 115 weeks)	HR (95%CI) CTD: 0.58 (0.33 to 1.02)
Ambrisentan	Galie 2008 ARIES‐1 and ARIES‐2^57^	2 RCTs IPAH, CTD‐PAH, others^b^ 6MWD 150‐450 m WHO FC I‐IV	Ambrisentan po 5‐10 mg/d Ambrisentan po 2.5‐5 mg/d	Placebo Placebo	201 192	126 125	62 62	placebo‐corrected ∆6MWT at week 12	CTD range: 15 to 23 m
NO pathway									
Sildenafil	Badesch 2007^58^	Subgroup analysis of RCT CTD‐PAH^b^ 6MWD 100‐450 m WHO FC II‐IV	Sildenafil po 20‐40‐80 mg 3x/d	Placebo	84	0	84 (38)	Placebo‐corrected Δ6MWT at week 12 20 mg, 40 mg or 80 mg	CTD 20 mg: 55 m (95%CI: 24 to 85 m), *P* < .01, 40 mg: 49 m (95%CI: 19 to 80 m), *P* < .01, 80 mg: 28 m (95%CI: −14 to 71 m), *P* = NS
Tadalafil	Galie 2009 PHIRST^59^	RCT, IPAH, CTD‐PAH, others^b^ 6MWD 150‐450 m WHO FC I‐IV	Tadalafil po 2.5‐10‐20‐40 mg/d	Placebo	405	247	95	Placebo‐corrected Δ6MWT at week 16 2.5 mg, 10 mg, 20 mg, 40 mg	CTD 2.5 mg: 18 m (95%CI: −27 to 63 m), 10 mg: 22 m (95% CI: −13 to 56 m), 20 mg: 50 m (95% CI: 16 to 83 m), 40 mg: 49 m (95%CI: 15 to 83 m)
Riociguat	Ghofrani 2013 PATENT‐1^60^	RCT, IPAH, CTD‐PAH, others^b^ 6MWT 150‐450 m WHO FC I‐IV	Riociguat po 1.5‐2.5 mg 3x/d	Placebo	443	272	111	Placebo‐corrected Δ6MWT at week 12 2.5 mg 3x/d	CTD 27 m (95%CI: −7 to 61 m)
Combination therapy									
Ambrisentan and Tadalafil	Coghlan 2017 AMBITION^38^	Subgroup analysis of RCT, CTD‐PAH^b^ WHO FC II‐III	Combination R/Po ambrisentan 10 mg + tadalafil 40 mg	MonoR/ Po ambrisentan 10 mg or Tadalafil 40 mg	187	0	187 (118)	combined mortality/morbidity primary endpoint (mean duration of treatment: 74 wk)	HR (95%CI) CTD: 0.43 (0.24‐0.77) SSc: 0.44 (0.22‐0.89)

Abbreviations: %, per cent; 6MWT, six‐minute walk test; CI, confidence interval; comp, comparator; CTD, connective tissue disease; CTD‐PAH or SSc‐PAH; ERA, endothelin‐1 receptor antagonist; FC, functional class; HR, hazard ratio; IPAH, idiopathic pulmonary arterial hypertension; IV, intravenous; m, metre; mg, milligram; N, number of; NA, not available; NO, nitric oxide; NS, not significant; NYHA, New York Heart Association; others, other causes of PAH than IPAH; PAH, pulmonary arterial hypertension; po, per oral; RCT, randomized controlled trial; SC, subcutaneous; SSc, systemic sclerosis; WHO, World Health Organization; Δ, delta.

^a^ PAH defined as mean pulmonary arterial pressure ≥35 mm Hg.

^b^ PAH defined as mean pulmonary arterial pressure ≥25 mm Hg.

Following the international guidelines, in patients with CTD‐PAH with NYHA FC II or III, initial oral combination therapy is started (ERA + PDE5‐i), while those in NYHA FC IV are treated with initial combination therapy including parenteral prostacyclin (epoprostenol). There is no evidence of a favourable effect of treatment with immunosuppression in SSc‐associated PAH. However, treatment with immunosuppressive drugs, consisting of six monthly intravenous pulses of cyclophosphamide and a tapering dosage of prednisolone in SLE and MCTD‐associated PAH has resulted in a clinical response in 50% of 16 patients.^40^ Furthermore, the French nationwide SLE‐PAH study showed a trend towards better survival in patients who received hydroxychloroquine.^20^

## 8 TREAT TO TARGET

Risk status for mortality of Idiopathic PAH patients can be assessed by a comprehensive tool described by the ESC and the ERS guidelines.^33^, ^41^ Risk criteria are based on the presence of clinical signs of right heart failure, progression of symptoms, the WHO functional class, the 6MWT, parameters of cardiopulmonary exercise testing, NT‐pro‐BNP level, presence of right atrial dilatation and pericardial effusion on imaging, and (invasive) haemodynamics. For each criterium, values are set to assign a low, intermediate or high risk of estimated mortality.^41^, ^42^, ^43^ The average score of the number of low‐risk criteria at baseline and during the first follow‐up discriminates the risk of death or lung transplantation and enables classification of patients as low (<5%), intermediate (5%‐10%) or high risk (>10%) for an estimated 1‐year mortality. Several studies investigated the validity of this scoring system by either using noninvasive criteria only or a combination of the noninvasive and invasive criteria.^43^, ^44^ In these studies, it became clear that this scoring system is valid and that the quantification of the number of low‐risk criteria present accurately predicts transplant‐free survival in PAH (Table 4). Changes in risk from initial evaluation to first follow‐up also predict survival. Although PAH associated with CTD is a more progressive disease as idiopathic PAH, this risk stratification tool is valid for PAH‐CTD as well.^42^ This instrument may help the clinician to intensify treatment by adding a third treatment modality and/or to start parenteral treatment with prostacyclin analogues if indicated. Of note is that lung transplantation is an option for eligible patients with CTDs as well.^45^

**Table 4:** Risk assessment in pulmonary arterial hypertension

Determinants of prognosis^a^ (estimated 1‐year mortality	Low risk < 5%	Intermediate risk 5%‐10%	High risk > 10%
Clinical signs of right heart failure	Absent	Absent	Present
Progression of symptoms	No	Slow	Rapid
Syncope	No	Occasional syncope^b^	Repeated syncope^c^
WHO Functional Class	I‐II	III	IV
6‐minute walking distance	>440 m	165‐440 m	<165 m
Cardiopulmonary exercise test	Peak VO_2_ > 15 mL/min/kg (>65% predicted) Ve/VCO_2_‐slope < 36	Peak VO_2_ 11 ‐ 15 mL/min/kg (35%‐65% predicted) Ve/VCO_2_‐slope 36 ‐ 44.9	Peak VO_2_ < 11 mL/min/kg (<35% predicted) Ve/VCO_2_‐slope ≥ 45
NT‐pro‐BNP plasma levels	BNP < 50 ng/L NT‐pro‐BNP < 300 ng/L	BNP 50‐300 ng/L NT‐pro‐BNP 300‐1400 ng/L	NT‐pro‐BNP > 1400 ng/L
Imaging (echo, CMR)	RA area < 18 cm^2^ No pericardial effusion	RA area 18‐26 cm^2^ No or minimal pericardial effusion	RA area > 26 cm^2^ Pericardial effusion XX
Haemodynamics	RAP < 8 mm Hg CI ≥ 2.5 L/min SvO_2_ > 65%	RAP 8−14 mm Hg CI 2.0‐2.4 L/min SvO_2_ 60%‐65%	RAP > 14 mm Hg CI ≤ 2.0 L/min SvO_2_ ≤ 60%

Abbreviations: 6MWD, 6‐minute walking distance; BNP ¼, brain natriuretic peptide; CI, cardiac index; CMR, cardiac magnetic resonance; NT‐pro‐BNP, N‐terminal pro‐brain natriuretic peptide; pred., predicted; RA, right atrium; RAP, right atrial pressure; SvO2, mixed venous oxygen saturation; VE/VCO2, ventilatory equivalents for carbon dioxide; VO2, oxygen consumption; WHO, World Health Organization.

^a^ Most of the proposed variables and cut‐off values are based on expert opinion. They may provide prognostic information and may be used to guide therapeutic decisions, but application to individual patients must be done carefully. One must also note that most of these variables have been validated mostly for idiopathic PAH and the cut‐off levels used above may not necessarily apply to other forms of PAH. Furthermore, the use of approved therapies and their influence on the variables should be considered in the evaluation of the risk.

^b^ Occasional syncope during brisk or heavy exercise, or occasional orthostatic syncope in an otherwise stable patient.

^c^ Repeated episodes of syncope, even with little or regular physical activity. Adapted from the ESC/ERS guidelines on pulmonary hypertension.^1^

## 9 UNMET NEEDS

Pulmonary arterial hypertension is a well‐known complication in CTDs, particularly in SSc. Ten per cent of the SSc patients will develop PAH during lifetime. However, diagnosing PAH is challenging since SSc patients may have group 1 PAH, group 2 PH secondary to left heart disease (due to myocardial fibrosis), group 3 PH secondary to interstitial lung disease, group 4 PH (CTEPH), pulmonary veno‐occlusive disease or a combination as shown in Table 2.^1^, ^46^ Although the number of treatments is increasing also for PAH‐CTD, mortality in especially SSc‐PAH remains high, with lower survival rates and lower response rate of the PAH‐specific therapy as compared to idiopathic PAH.^19^

In CTDs in general, patients suffer from different limitations and morbidity due to their disease and often experience alternating periods of more and less disease activity resulting in fatigue and reduced exercise tolerance. As a result, complaints of constitutional symptoms do not prompt the patients to seek medical help. It is therefore challenging to make an early diagnosis of PH in patients with CTDs. The 6th world symposium on PH task force proposed to change the haemodynamic definition of PH. A mean PAP > 20 mm Hg and PVR ≥ 3 Woods Unit instead of mean PAP ≥ 25 mm Hg. ^1^, ^5^ Although patients with borderline PAP (mean PAP 21‐24 mm Hg) did not need any PAH‐specific treatment, the 2015 ESC/ERS guidelines recommended close monitoring of these patients to detect the possible progression to PH, because of a higher risk of PAH.^1^ Many patients with borderline PAP have a mild stage of pulmonary vasculopathy and are at increased risk for developing PH.^47^, ^48^, ^49^, ^50^ In SSc, PAH is known as a complication with high prevalence, late diagnosis and high mortality. In the last decade, progression has been made in screening purposes for PAH in SSc, in organization of expert centres for PAH and SSc and in the importance of early start of initial combination therapy.

Another challenging aspect of PAH‐CTD is that patients often suffer from a combination of different PH groups such as groups 1, 2 and 3 in one patient, necessitating an even more tailored treatment schedule as in idiopathic PAH patients. However, in clinical trials patients with a combination of causes for PH are excluded and thus treatment of these patients is not evidence based. In our opinion, the main objective for future research should aim to improve prognosis in this vulnerable group by focusing both on morbidity and mortality. Furthermore, translational research on vascular biomarkers and the use of nailfold capillaroscopy could help to define patients at greater risk for the development of PAH and could make personalized screening programmes possible.

## CONFLICT OF INTEREST

The authors declare no conflict of interest and no funding for this manuscript.
